# Urban developmental environments alter tadpole phenotypes depending on origin

**DOI:** 10.1111/1365-2656.70071

**Published:** 2025-06-29

**Authors:** Andrew D. Cronin, Judith A. H. Smit, Jacintha Ellers, Wouter Halfwerk

**Affiliations:** ^1^ Amsterdam Institute for Life and Environment, Section Ecology and Evolution Vrije Universiteit Amsterdam Amsterdam The Netherlands; ^2^ Smithsonian Tropical Research Institute Balboa Ancón Panama

**Keywords:** adaptation, *Engystomops pustulosus*, larval development, phenotypic plasticity, urban ecology and evolution

## Abstract

1. Urbanisation is rapidly altering environments with severe impacts on life on earth. While impacts from urbanisation are widespread, organisms with metamorphic life cycles are particularly likely to be affected, as they often undergo a major shift in lifestyle and habitat across life stages. Early life stages also likely undergo exceptionally high selection due to elevated mortality rates. Despite the potential importance of these early stages, we often lack insight into how urban conditions influence individuals early in their development.
2. This study examined how exposure to urban environmental conditions alters developmental phenotypes in interaction with origin. We studied túngara frog tadpoles (*Engystomops pustulosus*) from urban and forest populations and conducted a reciprocal transplant between forest and urban treatments.
3. Wild collected breeding pairs from urban and forest populations created clutches produced in the lab. Clutches were then split in two and exposed for 2 weeks to urban or forest developmental conditions. Tadpole developmental timing, morphology and vigilance behaviour were subsequently measured to assess the effects of origin and treatment, as well as their interaction.
4. Urban developmental environments differ in water quality, are warmer, and have fewer predators compared to forest environments. Our findings demonstrate that tadpoles originating from urban populations have a faster developmental rate regardless of the environmental treatment. Tadpoles exposed to the urban environment have constrained growth compared to tadpoles exposed to the forest environment, independent of origin. Additionally, we find that urban origin tadpoles demonstrate increased behavioural plasticity in response to disturbance compared to forest origin tadpoles.
5. Our results show effects of urban conditions on developmental phenotypes both within and across generations, most likely as an adaptive response to higher aquatic temperature regimes in urban areas. Divergence in juvenile growth and development may underlie divergence in adult phenotypes typical of adult túngara frogs found in urban and forest areas. These findings demonstrate the need to integrate early developmental stages to fully understand the evolutionary and ecological implications of urbanisation.

## Graphical abstract

Urbanisation has rapidly altered the ecological and evolutionary contexts for many species. Using a reciprocal transplant experiment with frog larvae, we find that urban environments can alter morphological, developmental and behavioural traits at both ecological and evolutionary scales.

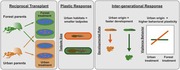

## Resumen

1. La urbanización está alterando rápidamente los entornos con graves impactos sobre la vida en la Tierra. Si bien los impactos de la urbanización son generalizados, los organismos con ciclos de vida metamórficos son particularmente propensos a verse afectados, ya que a menudo experimentan un cambio importante en el estilo de vida y el hábitat a lo largo de las etapas de la vida. Las primeras etapas de la vida también probablemente experimentan una selección excepcionalmente alta debido a las elevadas tasas de mortalidad. A pesar de la importancia potencial de estas primeras etapas, a menudo carecemos de conocimientos sobre cómo las condiciones urbanas afectan a los individuos al principio de su desarrollo.
2. Este estudio examinó cómo el estar expuesto a las condiciones ambientales urbanas altera los fenotipos de desarrollo en interacción con el origen. Estudiamos renacuajos de rana túngara (Engystomops pustulosus) de poblaciones urbanas y forestales y realizamos un trasplante recíproco entre tratamientos forestales y urbanos.
3. Las parejas reproductoras recolectadas en la naturaleza de poblaciones urbanas y forestales crearon nidadas producidas en el laboratorio. Luego, las nidadas se dividieron en dos y se expusieron durante dos semanas a condiciones de desarrollo urbanas o forestales. Posteriormente se midieron el tiempo de desarrollo, la morfología y el comportamiento de vigilancia de los renacuajos para evaluar los efectos del origen de los renacuajos y el tratamiento al que fueron expuestos, y también la interacción de estos dos.
4. Los entornos de desarrollo urbano difieren en la calidad del agua, son más cálidos y tienen menos depredadores en comparación con los entornos forestales. Nuestros hallazgos demuestran que los renacuajos originarios de poblaciones urbanas tienen una tasa de desarrollo más rápida independientemente del tratamiento ambiental. Los renacuajos expuestos al entorno urbano tienen un crecimiento limitado en comparación con los renacuajos expuestos al entorno forestal, independientemente del origen. Además, encontramos que los renacuajos de origen urbano demuestran una mayor plasticidad conductual en respuesta a las perturbaciones en comparación con los renacuajos de origen forestal.
5. Nuestros resultados muestran efectos de las condiciones urbanas en los fenotipos de desarrollo tanto dentro como entre generaciones, muy probablemente como una respuesta adaptativa a regímenes de temperatura acuática más altos en áreas urbanas. La divergencia en el crecimiento y desarrollo juvenil puede ser la base de la divergencia en los fenotipos adultos típicos de las ranas túngara adultas que se encuentran en áreas urbanas y forestales. Estos hallazgos demuestran la necesidad de integrar las primeras etapas del desarrollo para comprender plenamente las implicaciones evolutivas y ecológicas de la urbanización.

## 1 INTRODUCTION

Urbanisation represents a major change in the environment, altering abiotic and biotic conditions (Alberti, 2015; Cronin et al., 2022). Urban environments are characterised by increased sensory and chemical pollution, altered biological communities and increased temperatures, culminating in novel environmental conditions for many species (Niemelä, 2011). Populations of diverse taxa inhabiting urban environments exhibit phenotypic differences from non‐urban populations in adults (reviewed in Szulkin et al., 2020). Increasing focus on the evolutionary and ecological consequences of urbanisation has led to a better understanding of how and why these differences might arise, and whether phenotypic changes are adaptive (Campbell‐Staton et al., 2020; Halfwerk et al., 2019; Winchell et al., 2023). While some studies have examined entire lifecycles of individuals (e.g. Brans & De Meester, 2018; Charmantier et al., 2017), in many cases, our understanding is incomplete, with most studies looking at adaptations in adult phenotypes for explanations (Lambert et al., 2021). Differences expressed in urban adults could for example, be partially or entirely explained by plasticity or selection arising during early developmental stages (Corsini et al., 2021; Ouyang et al., 2019; Salmón et al., 2016), which may be particularly relevant when larval and adult stages are part of a complex life cycle, as is the case with amphibians. Or differences arising at early life stages could go undetected, for example, in cases with selective mortality of juveniles.

Influences of environmental variation on phenotypes during early development (i.e. developmental plasticity) are ubiquitous across taxa (Sultan, 2021; West‐Eberhard, 2003) and can cause an immediate, intragenerational effect of exposure to urban conditions (Ouyang et al., 2019). Some observed changes are temporary, while others are irreversible, leading to lasting phenotypic differences between individuals or populations experiencing varying conditions. This developmental plasticity may be an adaptive, non‐adaptive (inherently biophysical) or even maladaptive response to the current environmental conditions. Regardless of the underlying processes, these organismal responses to varying environmental developmental conditions may have significant carry‐over effects (Gomez‐Mestre et al., 2010; Johansson et al., 2010; Lee et al., 2012; Yagi & Green, 2018), suggesting that many phenotypic differences found between adults in urban and non‐urban populations may be largely due to plastic, intragenerational processes (Lambert et al., 2021).

Additionally, urban environmental conditions can change selection pressures, which consequently can alter developmental phenotypes through an inter‐generational effect. Although selection has the potential to act on all stages of life, it typically does so asymmetrically. During early life stages, organisms are typically smaller and less mobile, leading to elevated mortality during early phases of development, an effect particularly pronounced in species with large clutches and without parental care (Gosselin & Qian, 1997; Martin et al., 2018). Consequently, traits expressed during early development, whether behavioural, morphological or related to developmental timing, are highly consequential for individual fitness. Selection imposed by urban conditions may therefore be most pronounced during early stages, requiring examination of any potentially urban‐adapted phenotypes through a developmental lens.

Genetic and plastic effects can also be tightly intertwined if selection changes the developmental plasticity response itself, thereby changing the ability to sense and respond to environmental change (i.e. the degree of plasticity). Interestingly, plasticity is theorised and shown to be under stronger selection in populations and species occupying regions with high spatial and/or temporal heterogeneity (Gomez‐Mestre & Jovani, 2013; Hendry, 2016), a trademark of urban environments. Additionally, urban areas are characterised by many novel conditions, and increased developmental plasticity can facilitate the transition to a new adaptive peak in a fitness landscape altered by this novelty (i.e. via the ‘Baldwin effect’; Baldwin, 1896; Crispo, 2007; Snell‐Rood & Ehlman, 2021). As developmentally plastic traits can have significant and lasting consequences, variation in plasticity between urban and forest populations may facilitate or constrain adaptations in both early and late life stages.

To address our limited understanding of the implications of urban environments on developmental traits, we characterised urban and forest developmental sites of túngara frogs (*Engystomops pustulosus*), a model species for sexual selection with known phenotypic differentiation in response to urbanisation in adults (Halfwerk et al., 2019). We conducted a reciprocal transplant experiment to assess the morphology, development and vigilance behaviour of urban and forest túngara frog tadpoles. Due to the high heterogeneity of urban environments (Band et al., 2005), we predicted that urban origin tadpoles would display a higher degree of plasticity compared to forest origin tadpoles. Hence, we anticipated the presence of origin × treatment interactions, with forest origin tadpole morphological, developmental and behavioural responses to be relatively similar when comparing tadpoles raised in either forest or urban treatments, whereas urban origin tadpoles would show larger differences between urban and forest developmental treatments. Additionally, due to the urban heat island effect, we predicted that urban developmental environments would be warmer, potentially leading to faster development in the urban treatment (Albecker et al., 2023; Sinai et al., 2022). However, due to a general lack of prior knowledge concerning other aspects of the developmental environments of either urban and forest túngara tadpoles (e.g. predator abundance), we did not pose robust hypotheses about the direction of the specific treatment and origin effects.

## 2 MATERIALS AND METHODS

**FIGURE 1:**
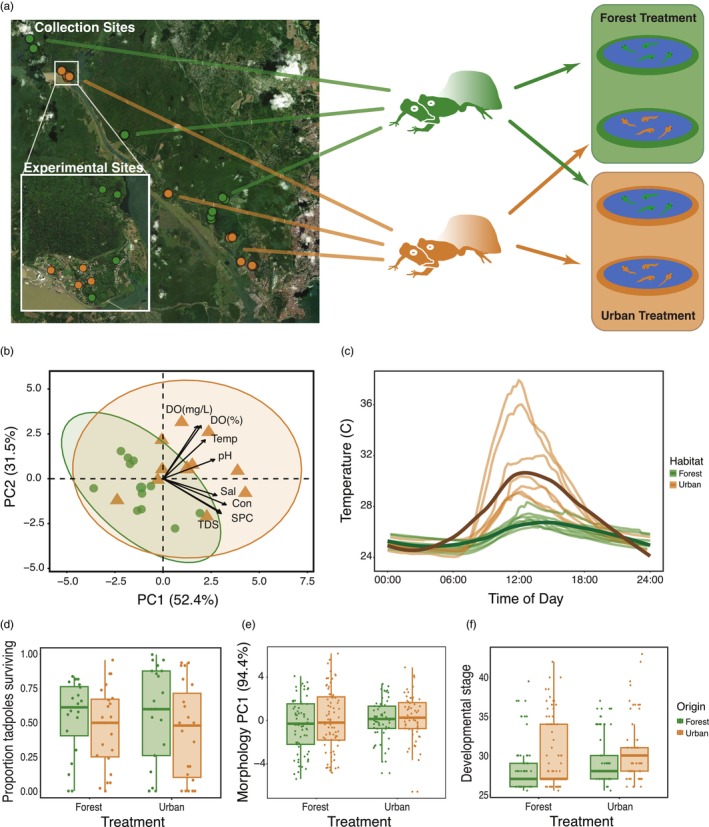
(a) (left) collection sites of parental pairs, with inset showing experimental sites. Green dots represent forest sites, and brown dots represent urban sites. (Right) clutches from urban and forest tadpoles were split, and each clutch was exposed to both treatments. (b,c) Urban and forest developmental conditions. Biplot showing PCA of water quality measures of natural breeding sites in the field with loadings (*n* = 21 collection sites from 9 locations) (loadings: DO(mg/L)‐dissolved oxygen (mg/L), DO(%)‐dissolved oxygen (% saturation), Temp‐temperature, pH‐pH, Sal‐salinity, Con‐conductivity, SPC‐specific conductance, TDS‐total dissolved solids) (b) and water temperatures of experimental developmental sites (*n* = 8 experimental sites) (c). (d–f) Tadpole responses based on origin and experimental treatment. Tadpole survivorship (*n* = 87 split clutches) (d), morphology (*n* = 261 individuals from 74 split clutches) (e), and developmental stage after 14 days of experimental exposure (*n* = 294 individuals from 76 split clutches) (f). See main text for statistics.

Túngara males and females clasped together in preparation to mate, a process known as amplexus, were collected from 29 ephemeral water bodies (15 forest and 14 urban) where adults mate and tadpoles develop (referred to hereafter as ‘collection sites’) from 5 forest and 4 urban locations around Panama City, Soberanía National Park and Camino de Cruces National Park, in Panamá. All experiments were conducted in and around Gamboa, Panamá (Figure 1a, Table S1). There were four rounds of experiments, all of which took place between September and November 2021 under the ethical approval of the Smithsonian Tropical Research Institute (STRI) (IACUC permit: 2019‐0301‐2022) and with collection permits obtained from the Autoridad Nacional Ambiente de Panamá (SE/A‐31‐2020).

### 2.1 Field methods

Collection of breeding pairs between urban and forest sites was balanced each night. Water samples (1 L) and GPS‐coordinates were taken at each collection site. To characterise collection sites, noise levels were measured using a Sound Pressure Level meter (Model: SL‐200, Voltcraft, Germany) and night light levels were measured with a lux meter (Model: HT309, HT Instruments, Italy). We also measured water quality, including temperature, pH, conductivity, total dissolved solids, dissolved oxygen and salinity with a YSI Professional Plus Multiparameter meter (Model: 6050000, YSI Incorporated, Ohio, USA). Because equipment could not be left in the field due to concerns of property loss, one measurement of water quality per collection site was taken. Canopy cover was quantified by approximating the percentage of cover directly over the collection site (Figure S1). Urbanisation scores were calculated using the ‘UrbanisationScore’ software (Seress et al., 2014), based on GPS‐coordinates of collection sites, to verify the classification of urban (mean = 2.05; SD = 1.30) and forest sites (mean = −1.50; SD = 0.54; Table S1). Measurement of sensory conditions further supported our classification of urban and forest sites, with urban collection sites having higher lux values compared to forest collection sites (linear mixed model (LMM): *n* = 29; *χ* ^2^ = 8.1209; *p*_adjusted_ = 0.02; Figure S2, Table S4).

### 2.2 Reciprocal transplant procedure

Breeding pairs (23 from urban sites and 23 from forest sites; Table S1) were brought back and placed in plastic containers (Ø 15 cm) with approximately 50 mL of dechlorinated tap water (Tetra ‘Aquasafe Plus’). Pairs were kept overnight, resulting in the production of a nest with fertilised eggs and were subsequently released back to their collection sites the following day. Each urban nest was experimentally matched with a forest nest collected the same day, creating a balanced design. Experimental manipulations began the day after collection by dividing the clutches evenly in two. In the lab, one half of the clutch was put in 500 mL of its natal water in a plastic container and the other half was put in 500 mL of water collected from the matched clutch of the other habitat type, creating full‐factorial treatment/origin combinations. Clutches were monitored regularly for hatching. Approximately 24 h after hatching began, we photographed each clutch, then randomly isolated 50 tadpoles for experimental use. For 14 out of 87 split clutches, the number of tadpoles within the split clutch was below 50 individuals, in which case we utilised all available tadpoles (mean = 31 ± 11 tadpoles, range = 4–49 tadpoles), leading to a total of 4085 tadpoles placed in experimental sites.

To closely simulate the developmental conditions for urban and forest tadpoles, we sampled water from parental collection sites. Additionally, we collected predators in each parental collection site by sweeping a small fish net within a roughly 0.6 m^2^ part of the breeding site for 5 seconds. Odonata larvae (dragonflies and damselflies) and beetles from the family Dytiscidae (predaceous diving beetles) were classified as predators based on published evidence or personal observation of predation on tadpoles. Sediment was collected by sampling a 0.02 m^2^ patch at each site. Sediment samples were collected until there was resistance felt by the collector. Sediment volume varied markedly between locations (mean = 228.80 mL, range = 0–1005.90 mL), but did not differ between urban and forest habitats (linear mixed model (LMM): *χ* ^2^ = 1.26; *p*_adjusted_ = 0.31; Figure S1, Table S4). In rare instances, the parental collection site had completely evaporated, in which case we sampled the nearest possible developmental site.

To standardise the dimensions of the puddles, and to control for water loss through the substrate, we created experimental developmental sites (Ø 50 cm plant drainage basins; hereafter referred to as ‘experimental sites’) in forested and urbanised areas around the town of Gamboa (Figure 1a). All experimental sites were selected to be nearby water bodies with túngara frog activity and, in urban areas, sites were placed away from human traffic to minimise the risk of direct human interference. Experimental sites were chosen to as closely as possible resemble the sensory conditions of collection sites (Figure S2, Tables S4 and S5). Experimental developmental sites (*n* = 87) were filled with the water, sediment and biotic community sampled from collection sites of the same habitat type, and finally the corresponding tadpoles (either urban or forest origin). Experimental sites were checked at least every other day to measure water depths and ensure that sites were undisturbed (Figure S3). Water temperature measurements of urban and forest experimental sites were collected every 15 minutes during the experiment (model: Tinytag 2 TPG‐4020, Gemini Data Loggers, United Kingdom).

### 2.3 Tadpole survivorship, development and morphology

After 14 days in the field, tadpoles were collected from the experimental sites and brought back to the lab. We selected 14 days of exposure as tadpoles of this species can begin to metamorphose around this time period under ideal circumstances (Hailey et al., 2006). All remaining tadpoles were first counted to determine survivorship. The following day, four tadpoles per clutch were randomly selected for developmental staging, morphological measurements and behaviour trials. To determine developmental stage and to collect morphological traits, tadpoles were placed in a small chamber filled with water and photographed from the side (Canon EOS 80D with EF‐S 60 mm Macro Lens). Developmental stages were based on an adapted Gosner staging table of a close relative, Physalaemus *biligonigerus* (for illustrations and photos of stages, see Chuliver & Fabrezi, 2019; Gosner, 1960). For 15 tadpoles, unequivocal determination of a particular developmental stage was not possible, in which case a range of developmental stages was assigned. In all cases, the range was only between two stages, and we selected the average of the range for statistical analyses.

Morphological traits were measured from photographs in ImageJ2 (version 2.9.0) (Rueden et al., 2017). We measured 5 traits: total length, body length, tail length, tail muscle depth at the base of the tail, maximum tail fin depth and body depth (derived from Relyea, 2000; Woodley et al., 2015; Figure S4). We conducted a principal component analysis on all log‐transformed traits. A subset was not included (*n* = 33) in the principal component analysis due to missing measurements (primarily due to tadpoles missing part of their tail).

### 2.4 Behavioural trials

Behavioural trials were conducted the day after tadpoles were brought to the lab between 8:00 and 16:00. To standardise the potential effects of hunger on tadpole behaviour, all tadpoles were fed fish flakes before behavioural trials. Tadpoles were isolated and placed within a plastic petri dish with dechlorinated tap water and were visually isolated from one another. Eight tadpoles were arranged on two trays (four per tray), with each tray attached to an electrodynamic exciter (model: EX 60 S, Visaton, Germany). Urban and forest treatment tadpoles from the same clutch were tested at the same time and were balanced across trays. After an acclimatisation period of 20 min, all tadpoles received the same one‐second vibrational stimulus, which was composed of broadband white noise, played via an mp3 player (model: MPS – 110NF, Conrad Electronics, Germany) connected to a stereo amplifier (model: SAP‐702, Conrad Electronics, Germany). The rationale behind this treatment is that this sudden and short exposure would change the water flow in a highly standardised way, mimicking a physical disturbance to the tadpole's environment (e.g. the presence of a predator or a falling object). After exposure to this stimulus, tadpoles demonstrated a freeze response before resuming movement. Freeze responses are a typical vigilance behaviour that is a widely displayed anti‐predator response in tadpoles (Lefcort, 1998; Stynoski & Noble, 2012; Takahara et al., 2012). Blind to both the treatment and origin of tadpoles, we scored the latency until the tadpole began to move again after this freeze response from video of behavioural trials (camera model: ELP‐USB4HDR01‐MFV, Shenzhen Ailipu Technology, China).

### 2.5 Data analyses

Statistics were run in R (version 4.2.2) (Team, 2022) and R Studio (version 2022.12.0 + 353) (Posit team, 2022). Mixed models were generated with packages *lme4* (version 1.1.31) (Bates et al., 2015) or *glmmTMB* (version 1.1.5) (Brooks et al., 2017). Graphs were created with *ggplot2* (version 3.4.1) (Wickham, 2016) using raw data.

We analysed variation in a suite of environmental variables from collection (water quality, predator abundance, sediment volume, canopy cover, noise, light) and experimental (noise, light, water depth and water temperature) developmental sites by fitting mixed models to each variable separately. In the case of water quality, a principal components analysis was performed on eight water quality variables, and the first and second principal components were used as response variables in analyses (Table S2). Linear mixed models with Gaussian distributions were fitted to all response variables, with the exception of predator abundance and water temperature. Due to overdispersion, predator abundance, which was measured as the total abundance of predators, was fitted with a negative binomial model. We also utilised non‐parametric Kruskal–Wallis tests for daytime and nighttime temperatures due to heteroscedasticity in these data. Models for analysis of environmental variables included habitat or treatment (urban/forest) as a fixed factor. For analyses of environmental variables at collection sites, sampling location was included as a random intercept, while analyses of environmental variables at experimental sites included site ID (see Tables S4 and S5 for full model structure). As we ran a total of 14 models to investigate environmental differences between urban and forest environments, increasing the possibility of type I error, we utilised the Benjamini and Hochberg method to adjust p‐values and control for false discovery rates (Benjamini & Hochberg, 1995).

We investigated the effects of origin (urban/forest), treatment (urban/forest) and their interaction by running six models on four tadpole response variables: survivorship, developmental rate, tadpole morphology and tadpole freeze responses. For tadpole morphology, we ran three separate models. The first model examining tadpole morphology excluded the developmental stage to examine if there was an overall effect of habitat or treatment on tadpole morphology. Two additional models were used to investigate tadpole morphology while taking into account developmental stage (see below for details). To account for any variation caused by the number of tadpoles at the beginning of the experiment, we added the number of starting tadpoles as a covariate for all models. Unless otherwise specified, the location of the collection site, experimental site and clutch ID were added as random intercept effects due to the experimental design. Each level of the random effects was given a unique identifier, allowing for explicit nesting of the random effects model structure. For developmental rate, tadpole morphology and tadpole freeze responses, split clutch ID was also included as a random intercept effect with a unique identifier for all levels, as multiple individuals were sampled from the same split clutch. The full model for developmental rate did not include the location of origin due to convergence issues. We also converted developmental rate data into a binary variable (above/below overall mean) due to heterogeneity of variance in the raw data and created a binomial generalised linear mixed model with a logit link function. The latency to movement measure from behavioural trials was log‐transformed prior to analysis to improve model fit. All other models were based on a gaussian distribution with an identity link function. Full model structures are presented in Table 1.

**TABLE 1:** Results of linear mixed models (LMM) and generalised linear mixed models (GLMM) for all tadpole trait models.

Response variable	Explanatory variable	Estimate	SE	*χ* ^2^	*p* value
Survivorship ~ origin × treatment + Initial tadpole # + (1|clutch) + (1|experimental site) + (1|location), family = betabinomial, link = logit					
Survivorship	Intercept	−0.08	0.90		
	Initial Tadpole #	0.00	0.02	0.01	0.921
	Origin × Treatment	0.41	0.52	0.63	0.428
	Origin	0.38	0.34	1.26	0.261
	Treatment	0.07	0.39	0.03	0.865
#tadpoles over mean Gosner stage ~ origin × treatment + Initial tadpole # + (1|clutch) + (1|split clutch) + (1|experimental site), family = binomial, link = logit					
Developmental Speed	Intercept	−1.58	3.36		
	Initial Tadpole #	−0.03	0.07	0.27	0.607
	Origin × Treatment	1.11	1.47	0.59	0.444
	**Origin**	**2.44**	**0.92**	**7.77**	**0.005**
	Treatment	1.10	2.18	0.26	0.610
PC1 ~ origin × treatment + Initial tadpole # + (1|clutch) + (1|split clutch) + (1|experimental site) + (1|location), family = gaussian					
Size	Intercept	−0.01	1.47		
	Initial Tadpole #	−0.01	0.02	0.13	0.716
	Origin × Treatment	−0.12	0.67	0.04	0.841
	Origin	0.73	0.56	1.86	0.173
	Treatment	0.36	1.27	0.11	0.735
PC1 ~ origin × treatment + Gosner stage + Initial tadpole # + (1|clutch) + (1|split clutch) + (1|experimental site) + (1|location), family = gaussian					
Size/stage (GS 25–28)	Intercept	−27.95	4.21		
	Initial Tadpole #	−0.02	0.02	1.10	0.294
	**Gosner Stage**	**1.03**	**0.15**	**39.30**	**<0.001**
	Origin × Treatment	−0.13	0.61	0.03	0.852
	Origin	−0.31	0.34	0.87	0.350
	Treatment	−0.18	0.61	0.11	0.738
Size/stage (GS 29–43)	Intercept	−8.02	1.40		
	Initial Tadpole #	0.01	0.02	0.46	0.499
	**Gosner Stage**	**0.30**	**0.03**	**81.61**	**<0.001**
	Origin × Treatment	0.15	0.45	0.13	0.723
	Origin	0.01	0.43	<0.01	0.999
	**Treatment**	**−0.62**	**0.22**	**4.92**	**0.027**
log10(response time) ~ origin × treatment + Initial tadpole # + (1|split clutch) + (1|clutch) + (1|location) + (1|Gosner stage) + (1|experimental site), family = gaussian					
Response time (log10(seconds)) with interaction	Intercept	1.37	0.21		
	Initial Tadpole #	0.01	<0.01	1.41	0.236
	**Origin** × **Treatment**	**0.22**	**0.10**	**4.06**	**0.044**
	Origin	−0.01	0.10	<0.01	0.947
	**Treatment**	**0.15**	**0.07**	**3.89**	**0.049**

*Note*: Models test the effects of tadpole origin (urban or forest), treatment (urban or forest), and their interaction on tadpole survival, developmental rate, tadpole size, and time to respond after induced freezing response. Interaction estimates and standard errors (SE) are taken from the full model. Other estimates and SE are taken from reduced models, excluding non‐significant interaction terms. Origin, treatment, and their interaction are fixed effects, whereas initial tadpole number and Gosner state are used as covariates. All levels of random intercept effects were given unique identifiers to allow for nesting. Bolded terms indicate significant effects (*p* < 0.05).

The morphological response variable was generated by incorporating the first principal component from our PCA, which explained 94.4% of the variance in our morphological data. Based on the high positive correlations of all morphological traits (Spearman rank correlation, all *r* > 0.87), we interpret these increasing PC1 values as increasing tadpole size (Figure S5, Table S3). Due to unequal numbers of tadpoles across different developmental stages resulting in significant heterogeneity of variance, we split the morphological data into two groups and ran separate models for each group. These groups were separated along the break point, where there was a clear change in slope, which occurred between Gosner Stage 28 and 29 (Gosner, 1960), determined with R package *segmented* (version 1.6.1) (Muggeo, 2008). As we were interested in examining the environmental and origin effects on the relationship between developmental stage and tadpole size, we ran models both with and without developmental stage as a covariate.

We incorporated a backwards selection approach for all model comparisons, utilising likelihood ratio tests. Model estimates were obtained from the most reduced model, with any statistically insignificant interaction terms excluded. All model assumptions were checked using the *DHARMa* package (version 0.4.6) (Hartig, 2021).

## 3 RESULTS

### 3.1 Urbanisation affects developmental environment

Urban and forest collection sites significantly differed in water quality based on a principal component analysis after correction for multiple comparisons (proportion of variance explained by PC1 = 0.52; linear mixed model [LMM]: *n* = 21; *χ* ^2^ = 6.77; *p*_adjusted_ = 0.03; Figure 1b, Tables S2 and S4). The loadings from the first principal component indicate that urban collection sites were characterised by higher pH, conductivity, salinity, total dissolved solids and temperature compared to forest sites. In addition, forest collection sites had more predators (generalised linear mixed model [GLMM]: *n* = 29; *χ* ^2^ = 7.97; *p*_adjusted_ = 0.02; Table S4). Predator communities were largely made up of predatory diving beetles (family Dytiscidae) and dragonfly larvae (order Odonata) (Figure S1).

Similar to the collection sites, daily temperatures of urban experimental sites were warmer (forest mean = 26.0°C; urban mean = 28.5°C; Kruskal–Wallis: *n* = 14; *χ* ^2^ = 9.8; *p*_adjusted_ = 0.01) and were also more variable (forest range = 23.4–31.6°C; urban range = 22.7–43.4°C). At night, differences in mean temperature vanished (Kruskal–Wallis: *n* = 14; *χ* ^2^ = 0.10; *p*_adjusted_ = 0.81; Figure 1c, Figure S3).

### 3.2 Effects of origin and treatment on developmental rate and body size

**FIGURE 2:**
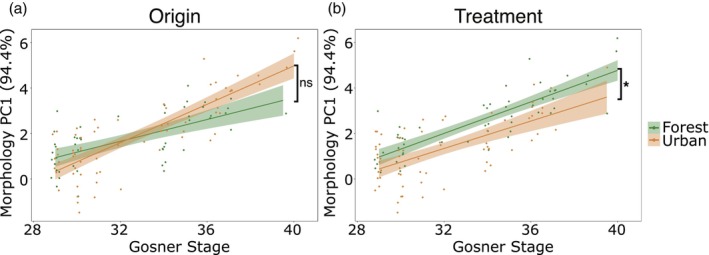
Tadpole size corrected for developmental stage based on origin (a) and treatment (b) (*n* = 261 individuals from 74 split clutches). See Table S3 for loadings for PC1. *p < 0.05. ns = not significant.

After 2 weeks of exposure to either urban or forest treatments, we found that tadpoles originating from urban sites developed at a faster rate (i.e. were at a later developmental stage after 14 days; Figure 1f) and contained a higher proportion of late Gosner stages compared to forest origin tadpoles (GLMM: *n* = 294 tadpoles from 76 split clutches; *χ* ^2^ = 7.77; *p* = 0.005; log odds estimate = 2.44; Table 1). The effect of urban and forest origin on developmental rate was not dependent on treatment (*χ* ^2^ = 0.59; *p* = 0.44), and there was no overall developmental treatment effect (*χ* ^2^ = 0.26; *p* = 0.61). As survivorship did not differ based on treatment (*χ* ^2^ = 0.03; *p* = 0.87), origin (*χ* ^2^ = 1.26; *p* = 0.26) or their interaction (GLMM: *n* = 87 split clutches; *χ* ^2^ = 0.63; *p* = 0.43), we can rule out density differences as an underlying mechanism of the observed increased developmental rate in urban origin tadpoles (Figure 1d). We found no overall differences between the urban and forest treatment (*n* = 261 tadpoles from 74 split clutches; *χ* ^2^ = 0.09; *p* = 0.77) or origin (*χ* ^2^ = 1.78; *p* = 0.18) on tadpole size based on the first principal component of the five measured morphological traits (Figure 1e, Table 1). When controlling for differences in developmental stage (i.e. adding Gosner stage as a covariate), however, tadpoles exposed to the urban treatment were smaller than forest treatment tadpoles (linear mixed model (LMM); *χ* ^2^ = 4.92, *p* = 0.03; estimate = − 0.62; Figure 2b, Table 1). We found no interaction between origin and treatment (*χ* ^2^ = 0.13; *p* = 0.72), and no effect of origin on stage‐controlled tadpole size (*χ* ^2^ = 0.003; *p* = 0.99; Figure 2a).

### 3.3 Developmental effects on behavioural plasticity

**FIGURE 3:**
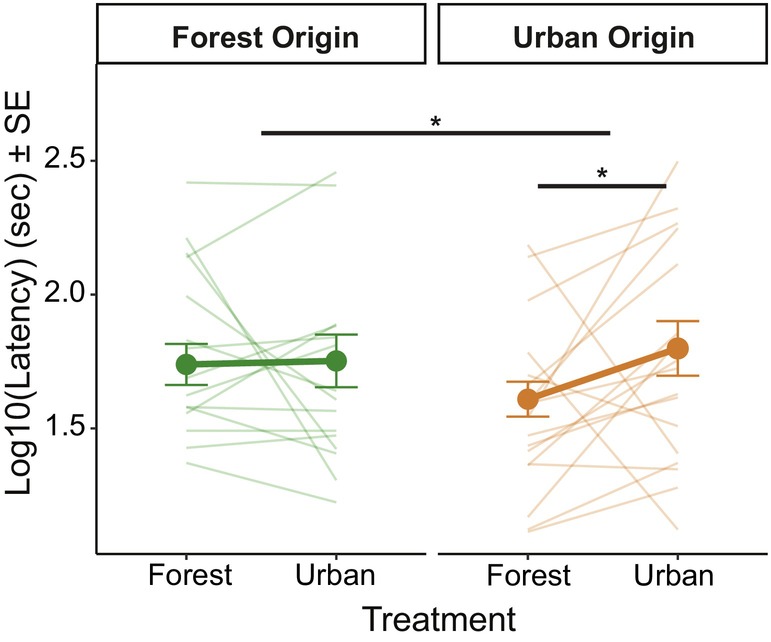
Vigilance behaviour (latency to resume movement in log seconds) in response to disruptive vibrational stimulus based on origin and treatment (*n* = 294 individuals from 76 split clutches). Thin lines connect the averages of tadpoles from a single clutch. Asterisks indicate statistical significance of interaction effects between treatment and origin (line between forest and urban origins), and of treatment effects within origins (line within forest or urban origins). Data points represent means ± standard error. *p < 0.05.

We tested tadpole vigilance responses to a disruptive vibrational stimulus and found a statistically significant interaction effect between treatment and origin (*n* = 294 tadpoles from 76 split clutches; LMM: *χ* ^2^ = 4.06; *p* = 0.04; Figure 3, Table 1), indicating that developmental treatment affected urban and forest origin tadpoles differently. Urban origin tadpoles took longer to resume activity after stimulus playback when raised in an urban environment compared to a forest environment (88% increase in latency) (estimate = −0.26, df = 7.93, *p* = 0.02). Forest origin tadpoles were behaviourally consistent between both treatment groups, only showing a negligible 6% increase in latency for tadpoles raised in the urban environment (estimate = −0.04; df = 8.11, *p* = 0.64).

## 4 DISCUSSION

Using a reciprocal transplant experiment, we document both developmental plasticity and origin effects on early life stages in the túngara frog. Urban developmental conditions led to smaller tadpole sizes than forest developmental conditions, showing a plastic response in body size to urbanisation. Changes in developmental rate were driven by inter‐generational processes, possibly resulting from a selective response in the different habitats of origin; tadpoles originating from urban populations overall developed at a faster rate than those from forest populations. We found no interaction between origin and treatment for body size or developmental rate, indicating that developmental plasticity for these traits did not differ based on origin. However, the vigilance of urban tadpoles in response to an aversive stimulus was highly dependent on their developmental conditions, showing plasticity in urban tadpoles, a pattern absent in forest tadpoles.

The urban treatment effect on body size showed that tadpoles reached smaller sizes per developmental stage, without any effect on developmental speed. In anurans, higher temperatures consistently lead to smaller body sizes (Albecker et al., 2023; Charbonnier & Vonesh, 2015), and daily temperatures were higher in the urban developmental environments for túngara frogs as well. Counterintuitively, average daily urban water temperatures were almost identical to optimal growing temperatures of túngara frogs (28.8°C) (Oyamaguchi et al., 2018), suggesting that urban frogs should grow faster based on average urban thermal conditions. However, temperatures greatly fluctuated in urban experimental sites, with periods of time when temperatures exceeded the thermal performance breadth and occasionally exceeded the critical thermal maximum for this species (Oyamaguchi et al., 2018). Additional environmental factors, like higher density, lower food availability and lowered water levels, often lead to reduced body size (Albecker et al., 2023). In the present study, we found no detectable differences between urban and forest development environments in any of these factors. Although results are mixed, some studies have noted an associated decrease in body size in response to exposure to predators (Perotti et al., 2016; Steiner & Van Buskirk, 2008). In this study, the larger body sizes per stage in the forest environment, which had higher levels of predation pressure, suggest that predation is unlikely to be a direct driver of size differences. Taken together, our findings suggest this plasticity in body size is likely a nonlinear response to a suite of environmental factors with temperature playing a primary role. Tests examining various environmental factors, including temperature, in isolation and in combination would provide a better understanding of the relationship between multiple environmental factors and tadpole size.

In the family Leptodactylidae, to which túngara frogs belong, about 50% of the variance in body size of adults can be explained by their body size at earlier developmental stages (Phung et al., 2020). Therefore, smaller tadpole body sizes found in urban conditions may partially explain the smaller body sizes found in adult urban males of this species (Halfwerk et al., 2019). Small tadpoles are likely already smaller at metamorphosis, which can also lead to reductions in fitness traits and survivorship (Cabrera‐Guzmán et al., 2013; Tarvin et al., 2015). There are also important longer‐term consequences of smaller body size, including impacts on reproductive success. For females, a smaller body size likely corresponds to a reduction in the size or number of eggs produced (Monroe et al., 2015). For males, a smaller body size may alter sexual signals, leading to less attractive signals with lower amplitude and higher frequencies (James et al., 2021; Muñoz et al., 2020). Reduced reproductive output of smaller males in túngara frogs is therefore likely, as females of this species show a robust preference for calls from larger males (Smit et al., 2019).

While the developmental environment can clearly play an important role in shaping various phenotypes of developing tadpoles, ancestral exposure to certain environments and selection pressures also shapes phenotypes (Auer et al., 2018; Des Roches et al., 2022). For many anuran species reproducing in highly ephemeral water bodies, including túngara frogs, developmental rate is highly consequential in terms of survival (Denver et al., 1998; Leips et al., 2000) and higher risks of desiccation often correspond to more rapid development (Laurila et al., 2002; Richter‐Boix et al., 2011). Elevated temperatures are also known to speed up the developmental process (Albecker et al., 2023), though whether this reflects an adaptive response or a consequence of the inherent biophysical properties of developing in warmer waters is unclear. Regardless, consistently higher daily water temperatures could facilitate increased canalisation of faster developmental rates in urban tadpoles. In general, increased urban temperatures represent one of the strongest known selection pressures on various species, and are clear evolutionary drivers of urban phenotypic change in species such as acorn ants (Martin et al., 2019, 2021), water fleas (Brans et al., 2017, 2018) and dandelions (Woudstra et al., 2024). Biotic conditions, including increased predation pressure, have also been implicated in increased rates of development, though evidence for this is mixed (Albecker et al., 2023; Relyea, 2007). In other species, decreased predation pressure, which we find in our urban developmental environments, is associated with a slowing of pace of life traits, including developmental timing (Auer et al., 2018; Reznick & Endler, 1982). Therefore, our findings suggest that selection pressures based on temperature (and/or desiccation risk) better explain the faster developmental rates in urban environments than the measured biotic factors. These observed inter‐generational differences could be explained by parental (non‐genetic or epi‐genetic) or genetic effects. As previously mentioned, adult males are consistently smaller in urban environments and these differences appear to be heritable (Smit, 2024). Therefore, it is conceivable that differences in developmental timing could represent a genetic change in urban populations.

Faster developmental rates, whether arising via adaptive or non‐adaptive mechanisms, mean that tadpoles of urban origin are able to leave their developmental environments sooner. In a species that develops in highly ephemeral water bodies, this may allow urban origin tadpoles to more successfully occupy developmental sites that are possibly at higher risk of desiccation or overheating. The ability to leave developmental sites more quickly may also mean that urban populations may be better able to cope with more variable rainfall patterns associated with shifting climatic conditions (van de Pol et al., 2017). Although sped up developmental rates may create opportunities for urban frogs to successfully operate in riskier developmental sites, this increased rate could lead to trade‐offs, specifically with body size. Surprisingly, we find no indication of this trade‐off in urban origin tadpoles, suggesting that these tadpoles may have evolved a way to mitigate this potential negative consequence. However, trade‐offs may still exist with other important fitness traits not measured in this study, such as immunocompetence (Rantala & Roff, 2005), lifespan (Lee et al., 2013) or reproductive output (Lee et al., 2012).

As urban environments tend to be characterised by both novel and heterogeneous environmental conditions, selection for increased plasticity is anticipated in these areas (Snell‐Rood & Ehlman, 2021). In our study, although urban environmental variables tended to show increased variation, we found that treatment effects were not dependent on origin for either developmental rate or body size. This lack of interaction suggests there is no difference in the degree of plasticity in morphology and developmental rate between urban and forest origin tadpoles. This apparent inconsistency with our predictions may be explained by several factors, including that differences in experimental conditions (such as temperature) may have been lower than laboratory studies showing changes in developmental rates. Also, although data is generally lacking for tropical species, it is currently thought that tropical species generally show less plasticity in their development than temperate species (Sinai et al., 2022). Many studies examining plasticity do so in response to single factors (e.g. heat or predation stress), whereas multiple potential sources of selection are simultaneously in operation in natural environments, possibly making direct comparisons to more controlled laboratory experiments challenging. Finally, the lack of differences in plasticity for morphological or developmental traits between urban and forest tadpoles could indicate that these traits are already maximally plastic.

While the specific mechanisms of increased behavioural plasticity are unclear (e.g. paternal effects or genetics), behavioural flexibility may be an important component allowing urban populations to deal with the heterogenous environmental and biotic urban mosaic. Vigilance behaviour can serve as an anti‐predator strategy or can be more broadly interpreted as a response to a physical disturbance in the environment. Urban environments are likely to be more heterogenous in a variety of environmental traits (Band et al., 2005), including in terms of disturbance. Previous studies have demonstrated positive selection on plasticity itself in more heterogenous environments (Edelaar et al., 2017; Lázaro‐Nogal et al., 2015; Merilä et al., 2004), suggesting that the behavioural plasticity seen in urban tadpoles may be adaptive. However, behavioural plasticity could also be neutral or maladaptive in more variable environments and should be further investigated by examining individual fitness. In this study, urban tadpoles began moving more quickly when raised in forested, compared to urban, environments, which was contrary to our expectations. We anticipated that urban environments would have increased disturbance (e.g. increased vibrational disturbance from construction or vehicles), causing urban tadpoles in urban environments to have the shortest freeze time, similar to vigilance behaviour in adults (Halfwerk et al., 2019). Our findings suggest that forested developmental environments have relatively higher levels of disturbance, perhaps due to more frequent disturbance by predators in these environments. Increased behavioural plasticity in urban populations has also been documented in other behaviours in adults of this species (Halfwerk et al., 2019). In general, behavioural phenotypes are thought to play an important role in facilitating the establishment and evolution of urban populations (Caspi et al., 2022), although behavioural changes may also weaken selection pressures, and ultimately slow down evolutionary change (i.e. the ‘Bogert effect’; Bogert, 1949; Huey et al., 2003; Muñoz, 2022). Although we are unable to directly relate larval to adult behavioural phenotypes in this species, behavioural traits can be consistent across metamorphosis (Bégué et al., 2023), suggesting that increased behavioural plasticity seen in urban adult túngara frogs may already be present in early life stages.

## 5 CONCLUSIONS

Tadpoles occupying urban areas are likely exposed to both immediate urban conditions and affected by past selection pressures, which lead to both a plasticity‐driven reduction in size, as well as an inter‐generational increase in developmental speed and behavioural plasticity. Such differences suggest that both selection during early developmental stages and environmental factors influencing development likely play a major role in phenotypic differentiation between urban and non‐urban populations. Additionally, our findings further support arguments that behaviour may play an important function in facilitating the establishment and evolution of urban populations. We therefore highlight the importance of integrating developmental traits into research on urban ecology and evolution to fully understand the potential environmental impacts, selection pressures, and evolutionary potential in urban areas. Such knowledge can not only further elucidate urban ecology and evolution but may also facilitate conservation methods to help the many species that are increasingly confronted with urbanisation worldwide (Lambert & Donihue, 2020).

## AUTHOR CONTRIBUTIONS

Andrew D. Cronin, Judith A. H. Smit, Jacintha Ellers and Wouter Halfwerk conceived the ideas and designed methodology; Andrew D. Cronin and Judith A. H. Smit collected the data; Andrew D. Cronin analysed the data; Andrew D. Cronin led the writing of the manuscript. All authors contributed critically to the drafts and gave final approval for publication.

## CONFLICT OF INTEREST STATEMENT

The authors declare they have no conflicts of interest.

## STATEMENT ON INCLUSION

Our study developed collaborations with scientists based in the country where the study was conducted. However, we recognise that we could have further integrated local scientists within our research during the course of this project. We plan to more closely collaborate with local scientists and local entities in future projects, starting in the research design phase.

## Supporting information


**Figure S1.** Environmental measures of collection sites in urban and forest environments.
**Figure S2.** (A, C) Sensory conditions of urban and forest natural collection sites where amplexed pairs were collected (*n* = 29 collection sites from 9 locations). (B, D) Sensory conditions of urban and forest experimental sites (*n* = 8 experimental sites).
**Figure S3.** (Left) Water depths of urban and forest experimental sites (*n* = 87 observations from 8 experimental sites). (Right) Average day time temperatures of experimental sites (*n* = 14 observations from 8 experimental sites).
**Figure S4.** Morphological measurements: (a) total length, (b) body length, (c) tail length, (d) body depth, (e) muscle depth, (f) tail depth.
**Figure S5.** Correlation plot of all measured morphological traits for all tadpoles (*n* = 261 tadpoles from 74 split clutches).
**Table S1.** Overview of collection locations, including the number of parental pairs collected per location.
**Table S2.** Loadings and explained variance for PC1‐PC3 examining the water chemistry of collection sites.
**Table S3.** Loadings and explained variance for PC1‐PC3 examining the morphological traits of tadpoles after 14 days of experimental exposure.
**Table S4.** Results of linear mixed models (LMM) and generalized linear mixed models (GLMM).
**Table S5.** Results of linear mixed models (LMM).

## Data Availability

Data and code are publicly available from the DataverseNL data repository https://doi.org/10.34894/ETBRET (Cronin et al., 2025).
