# Supplementary material for: Urban developmental environments alter tadpole phenotypes depending on origin
Source: J Anim Ecol. 2025 Jun 29;94(9):1707–19. doi: 10.1111/1365-2656.70071 (PMC12424279; doi:10.1111/1365-2656.70071)
Supplement: Supplementary file 1 — Figure S1. Environmental measures of collection sites in urban and forest environments. Figure S2. (A, C) Sensory conditions of urban and forest natural collection sites where amplexed pairs were collected (n = 29 collection sites from 9 locations). (B, D) Sensory conditions of urban and forest experimental sites (n = 8 experimental sites). Figure S3. (Left) Water depths of urban and forest experimental sites (n = 87 observations from 8 experimental sites). (Right) Average day time temperatures of experimental sites (n = 14 observations from 8 experimental sites). Figure S4. Morphological measurements: (a) total length, (b) body length, (c) tail length, (d) body depth, (e) muscle depth, (f) tail depth. Figure S5. Correlation plot of all measured morphological traits for all tadpoles (n = 261 tadpoles from 74 split clutches). Table S1. Overview of collection locations, including the number of parental pairs collected per location. Table S2. Loadings and explained variance for PC1‐PC3 examining the water chemistry of collection sites. Table S3. Loadings and explained variance for PC1‐PC3 examining the morphological traits of tadpoles after 14 days of experimental exposure. Table S4. Results of linear mixed models (LMM) and generalized linear mixed models (GLMM). Table S5. Results of linear mixed models (LMM). [file JANE-94-1707-s001.docx]

**Supplemental Materials: Urban developmental environments alter tadpole phenotypes depending on origin**


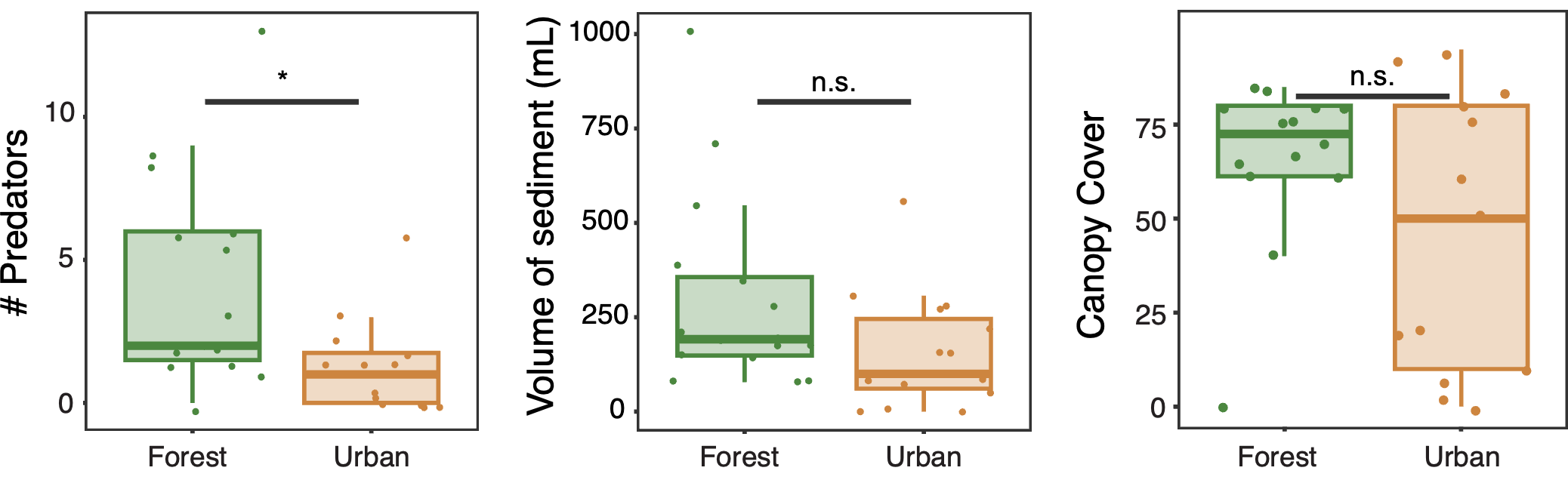
Fig. S1. Environmental measures of collection sites in urban and forest environments. *Left)* Predator abundance across urban and forest collection sites (n = 29 collection sites from 9 locations). *Middle)* Sediment volume across urban and forest collection sites (n = 29 collection sites from 9 locations). *Right)* Canopy cover for urban and forest natural collection sites (n = 27 sites from 8 locations). Significance is based on adjusted p-values using the false discovery rate method to account for testing fourteen different environmental response variables. See Table S4 for original and adjusted p-values. n.s. = not significant (*P* > 0.05). * *P* < 0.05.


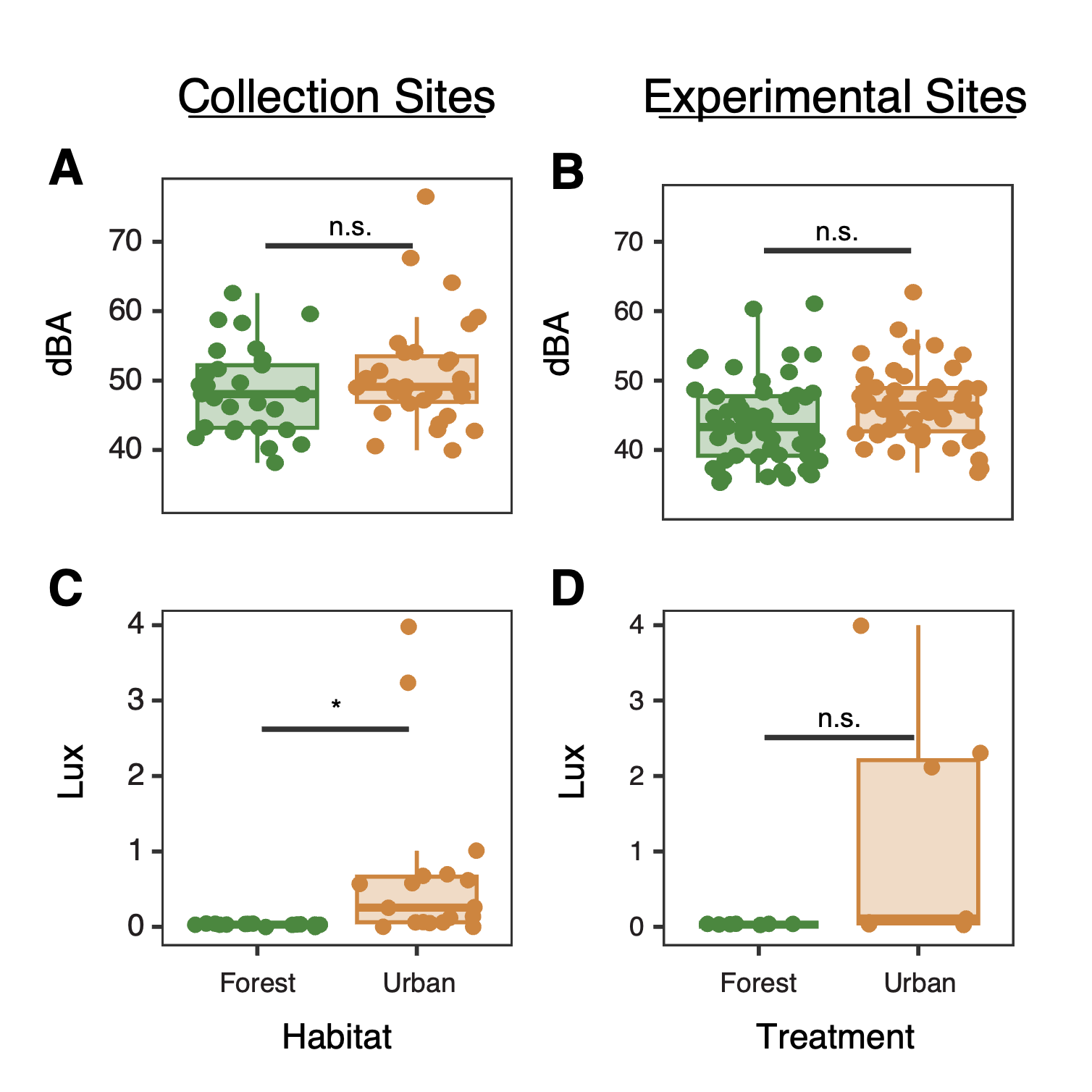
Fig. S2. (*A,C*) Sensory conditions of urban and forest natural collection sites where amplexed pairs were collected (n = 29 collection sites from 9 locations). (*B,D*) Sensory conditions of urban and forest experimental sites (n = 8 experimental sites). Significance is based on adjusted p-values using the false discovery rate method to account for testing fourteen different environmental response variables. See Tables S4 and S5 for original and adjusted p-values. n.s. = not significant (*P* > 0.05). * *P* < 0.05.


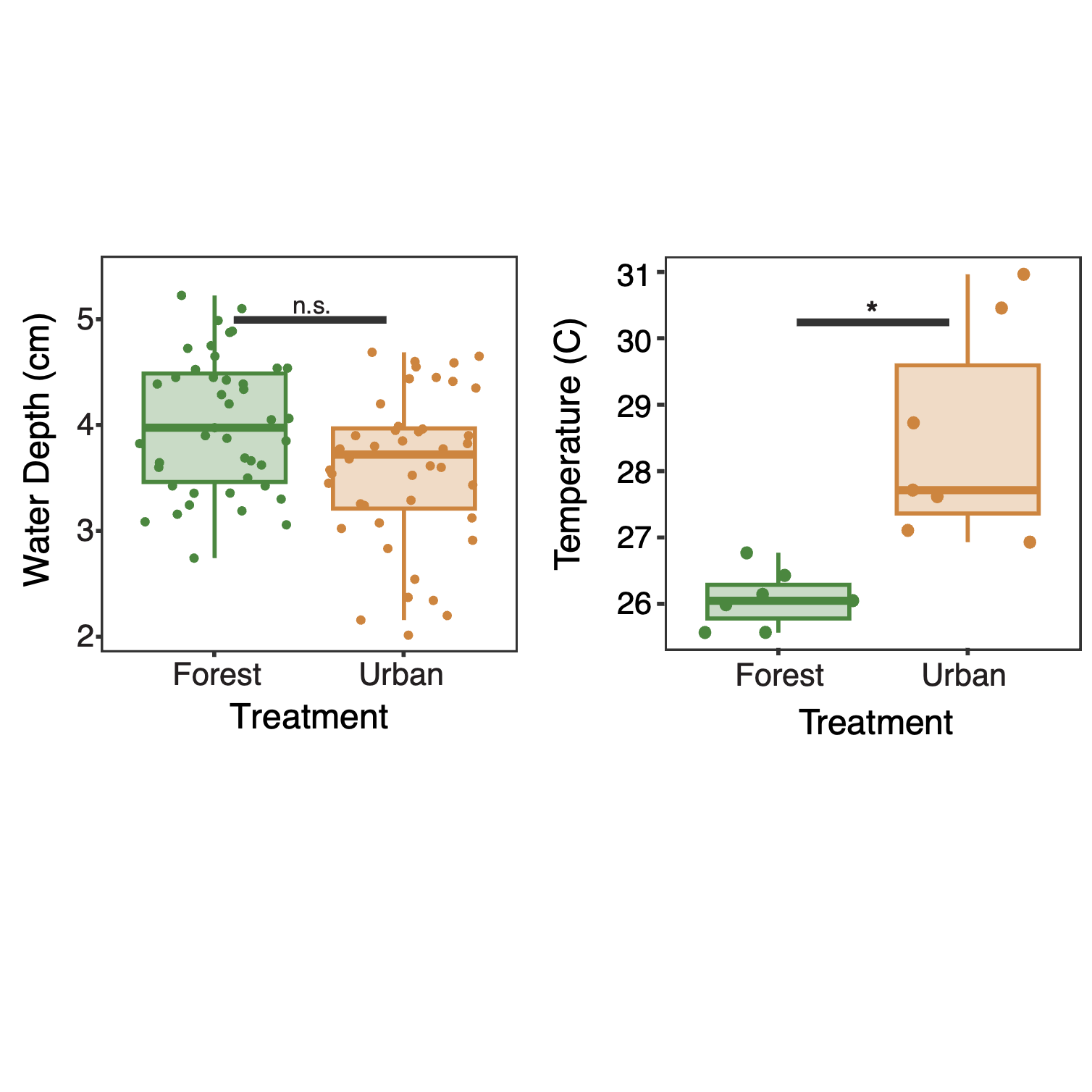
Fig. S3. *Left*) Water depths of urban and forest experimental sites (n = 87 observations from 8 experimental sites). *Right*) Average day time temperatures of experimental sites (n = 14 observations from 8 experimental sites) Significance is based on adjusted p-values using the false discovery rate method to account for testing fourteen different environmental response variables. See Table S5 for original and adjusted p-values. n.s. = not significant (P > 0.05). * *P* < 0.05.

Fig. S4. Morphological measurements: a = total length, b = body length, c = tail length, d = body depth, e = muscle depth, f = tail depth.

Fig. S5. Correlation plot of all measured morphological traits for all tadpoles (n = 261 tadpoles from 74 split clutches).

Table S1. Overview of collection locations, including the number of parental pairs collected per location.

Table S2. Loadings and explained variance for PC1-PC3 examining the water chemistry of collection sites.

Table S3. Loadings and explained variance for PC1-PC3 examining the morphological traits of tadpoles after 14 days of experimental exposure.

Table S4. Results of linear mixed models (LMM) and generalized linear mixed models (GLMM). Models test the environmental differences between forest and urban collection sites. Bolded terms indicate significant effects after adjustment of p-values (*P* < 0.05).

| **Response Variable** | **Explanatory Variable** | **Estimate** | **SE** | ***χ2*** | **P value** | **P_adjusted_** |
| --- | --- | --- | --- | --- | --- | --- |
| Water quality PC ~ habitat + (1 \| location), family = gaussian | | | | | |  |
| PC1 (52.43%) | Intercept | -1.10 | 0.58 |  |  |  |
|  | **Habitat** | **2.33** | **0.83** | **6.79** | **0.01** | **0.03** |
| PC2 (31.45%) | Intercept | -0.35 | 0.63 |  |  |  |
|  | Habitat | 0.93 | 0.90 | 1.32 | 0.25 | 0.31 |
| Predator abudance ~ habitat + (1 \| location), family = negative binomial | | | | | |  |
| *Total predator  abundance* | Intercept | 1.38 | 0.21 |  |  |  |
|  | **Habitat** | **-1.09** | **0.40** | **7.97** | **0.005** | **0.02** |
| Substrate volume ~ habitat + (1 \| location), family = guassian | | | | | |  |
| *Total  substrate (ml)* | Intercept | 304.07 | 86.35 |  |  |  |
|  | Habitat | -130.61 | 127.43 | 1.26 | 0.26 | 0.31 |
| Sensory environment ~ habitat + (1 \| location), family = guassian | | | | | |  |
| *Noise level (dBA)* | Intercept | 48.68 | 1.32 |  |  |  |
|  | Habitat | 2.27 | 1.83 | 1.56 | 0.21 | 0.30 |
| *Noise level (dBC)* | Intercept | 61.47 | 3.10 |  |  |  |
|  | Habitat | -0.14 | 4.55 | <0.001 | 0.99 | 0.99 |
| *Light level  (log10(lux))* | Intercept | -1.44 | 0.22 |  |  |  |
|  | **Habitat** | **1.01** | **0.31** | **8.12** | **0.004** | **0.02** |

Table S5. Results of linear mixed models (LMM). Models test the environmental differences between forest and urban experimental sites. Bolded terms indicate significant effects after adjustment of p-values (*P* < 0.05).

| **Response Variable** | **Explanatory Variable** | **Estimate** | **SE** | ***χ2*** | **P value** | **P_adjusted_** |
| --- | --- | --- | --- | --- | --- | --- |
| Sensory environment ~ treatment + (1 \| site), family = guassian | | | | | |  |
| *Noise level (dBA)* | Intercept | 45.09 | 0.78 |  |  |  |
|  | Treatment | 2.37 | 1.10 | 4.58 | 0.03 | 0.06 |
| *Noise level (dBC)* | Intercept | 52.88 | 1.27 |  |  |  |
|  | **Treatment** | **7.13** | **1.80** | **10.34** | **0.001** | **0.01** |
| *Light level  (log10(lux))* | Intercept | -1.52 | 0.35 |  |  |  |
|  | Treatment | 1.16 | 0.49 | 5.25 | 0.02 | 0.05 |
| Water depth ~ treatment + (1 \| site), family = guassian | | | | | |  |
| *Depth (cm)* | Intercept | 4.00 | 0.22 |  |  |  |
|  | Treatment | -0.43 | 0.31 | 2.20 | 0.14 | 0.23 |
